# The Theory and Practice of Medicine

**Published:** 1884-09

**Authors:** 


THE THEORY AND PRACTICE OF MEDICINE. *By* FREDERICK T. ROBERTS, M. D., B. SC., F. R. C. P. *With Illustrations. Fifth American Edition. 8 vo., pp. xiii, 1008. Philadelphia:* P. BLAKISTON, SON & CO., *1884. Chicago:* W. T. KEENER.

Professor Alfred Stillé, M. D., of the Medical Department of the University of Pennsylvania, has for years recommended "Roberts' Practice" to the members of his classes as in all respects the best *text-book* on the subject in the English language. A higher encomium, or more adequate guaranty of general excellence, could not be accorded a medical book. The fifth edition, materially modified by the discussions of the International Medical Congress in 1881, is an invaluable volume, which demands a prominent place in the library of every student and practitioner of medicine.

We note with pleasure the absence of the advertisement list usually found in American publications. W. W. J.

